# Pathogenetic and Clinical Aspects of Anti-Neutrophil Cytoplasmic Autoantibody-Associated Vasculitides

**DOI:** 10.3389/fimmu.2018.00680

**Published:** 2018-04-09

**Authors:** Peter Lamprecht, Anja Kerstein, Sebastian Klapa, Susanne Schinke, Christian M. Karsten, Xinhua Yu, Marc Ehlers, Jörg T. Epplen, Konstanze Holl-Ulrich, Thorsten Wiech, Kathrin Kalies, Tanja Lange, Martin Laudien, Tamas Laskay, Timo Gemoll, Udo Schumacher, Sebastian Ullrich, Hauke Busch, Saleh Ibrahim, Nicole Fischer, Katrin Hasselbacher, Ralph Pries, Frank Petersen, Gesche Weppner, Rudolf Manz, Jens Y. Humrich, Relana Nieberding, Gabriela Riemekasten, Antje Müller

**Affiliations:** ^1^Department of Rheumatology and Clinical Immunology, University of Lübeck, Lübeck, Germany; ^2^Institute for Systemic Inflammation Research, University of Lübeck, Lübeck, Germany; ^3^Xiamen-Borstel Joint Laboratory of Autoimmunity, Medical College of Xiamen University, Xiamen, China; ^4^Priority Area Asthma and Allergy, Research Center Borstel, Airway Research Center North (ARCN), German Center for Lung Research (DZL), Borstel, Germany; ^5^Laboratories of Immunology and Antibody Glycan Analysis, Institute for Nutrition Medicine, University of Lübeck and University Medical Center Schleswig Holstein, Lübeck, Germany; ^6^Department of Human Genetics, Ruhr-University, Bochum, Germany; ^7^University of Witten/Herdecke, ZBAF, Witten, Germany; ^8^Institute of Pathology, Cath. Mary’s Hospital, Hamburg, Germany; ^9^Institute of Pathology, University Hospital Hamburg-Eppendorf, Hamburg, Germany; ^10^Institute of Anatomy, University of Lübeck, Lübeck, Germany; ^11^Department of Otorhinolaryngology, Head and Neck Surgery, University of Kiel, Kiel, Germany; ^12^Department for Infectious Diseases and Microbiology, University of Lübeck, Lübeck, Germany; ^13^Department of Surgery, Section for Translational Surgical Oncology and Biobanking, University of Lübeck, University Medical Center Schleswig-Holstein, Lübeck, Germany; ^14^Institute of Anatomy and Experimental Morphology, Center for Experimental Medicine, University Cancer Center, University Medical Center Hamburg-Eppendorf, Hamburg, Germany; ^15^Medical Department 3, Gastroenterology/Rheumatology, Municipal Hospital Kiel, Kiel, Germany; ^16^Lübeck Institute of Experimental Dermatology, University of Lübeck, Lübeck, Germany; ^17^Institute of Medical Microbiology, Virology and Hygiene, University Medical Center Hamburg-Eppendorf, Hamburg, Germany; ^18^Department of Otorhinolaryngology, University of Lübeck, Lübeck, Germany

**Keywords:** anti-neutrophil cytoplasmic autoantibodies, anti-neutrophil cytoplasmic autoantibody vasculitides, microscopic polyangiitis, granulomatosis with polyangiitis, eosinophilic granulomatosis with polyangiitis

## Abstract

Anti-neutrophil cytoplasmic autoantibodies (ANCA) targeting proteinase 3 (PR3) and myeloperoxidase expressed by innate immune cells (neutrophils and monocytes) are salient diagnostic and pathogenic features of small vessel vasculitis, comprising granulomatosis with polyangiitis (GPA), microscopic polyangiitis, and eosinophilic GPA. Genetic studies suggest that ANCA-associated vasculitides (AAV) constitute separate diseases, which share common immunological and pathological features, but are otherwise heterogeneous. The successful therapeutic use of anti-CD20 antibodies emphasizes the prominent role of ANCA and possibly other autoantibodies in the pathogenesis of AAV. However, to elucidate causal effects in AAV, a better understanding of the complex interplay leading to the emergence of B lymphocytes that produce pathogenic ANCA remains a challenge. Different scenarios seem possible; e.g., the break of tolerance induced by a shift from non-pathogenic toward pathogenic autoantigen epitopes in inflamed tissue. This review gives a brief overview on current knowledge about genetic and epigenetic factors, barrier dysfunction and chronic non-resolving inflammation, necro-inflammatory auto-amplification of cellular death and inflammation, altered autoantigen presentation, alternative complement pathway activation, alterations within peripheral and inflamed tissue-residing T- and B-cell populations, ectopic lymphoid tissue neoformation, the characterization of PR3-specific T-cells, properties of ANCA, links between autoimmune disease and infection-triggered pathology, and animal models in AAV.

## Introduction

Anti-neutrophil cytoplasmic autoantibody (ANCA)-associated vasculitides (AAV) are classified into three distinct diseases based on clinical and pathological features: granulomatosis with polyangiitis (GPA, formerly Wegener’s granulomatosis), microscopic polyangiitis (MPA), and eosinophilic granulomatosis with polyangiitis (EGPA, Churg–Strauss syndrome). Etiology and pathogenesis of AAV are multifactorial (1, 2). The pathogenesis appears to be initiated by a combination of predisposing genetic and environmental factors, altered autoantigen presentation, ectopic lymphoid tissue neoformation, and imbalance of effector and regulatory B- and T-cells. Consequently, production of pathogenic autoantibodies originating from precursor natural autoantibodies results in ANCA-induced activation of neutrophils and monocytes with subsequent activation of the alternative complement pathway, vascular damage, and self-perpetuating non-resolving chronic inflammation (2–4). Herein, we briefly summarize current ideas, observations, and evidence on the pathogenesis of AAV.

## Clinical Manifestations

While each of the three AAV retains a unique clinical phenotype, many manifestations are shared among them owing to the systemic nature of the underlying small-vessel vasculitis, and, in GPA and EGPA, granulomatous inflammation. Thus, pulmonary–renal syndrome is the dominating clinical feature in GPA and MPA (5, 6). In EGPA, renal involvement is associated with positive ANCA-status (7, 8). Prodromes such as malaise, arthralgias, myalgias—and rhinitis and/or sinusitis in GPA and EGPA—often precede manifestations of the pulmonary–renal syndrome by weeks or months (5–7). Fulminant AAV is rare (9). EGPA patients typically have a long-standing history of asthma and allergic rhinitis (7, 10). Other organs frequently affected are peripheral and central nervous system, skin, gut, and heart (5–7). Laboratory findings show elevated markers of inflammation (3, 11). GPA is highly associated with proteinase 3 (PR3)-specific ANCA, whereas MPA and—less commonly—EGPA are associated with myeloperoxidase (MPO)-specific ANCA (12–14). The majority of AAV patients (approximately 80–90%) present with renal or other organ-threatening manifestations, i.e., generalized disease. Relapse is more common in GPA (15). Renal-limited AAV is less common. Fewer than 10% of patients have a localized phenotype restricted to the upper and/or lower respiratory tract or early-systemic phenotype without imminent organ failure with less frequently detected ANCA (5, 11, 12, 16, 17). Progression from localized to generalized GPA is rare (16).

Treatment is guided by severity of organ involvement and disease activity. Various cytotoxic immunosuppressants and the monoclonal anti-CD20 antibody rituximab are recommended for the induction and maintenance of remission (18, 19). While treatment options have improved the prognosis of AAV, therapy de-escalation still carries an immanent risk of relapse (20). Major causes of death are vasculitis and infections (21). Optimizing treatment strategies according to prognostic subsets, autoantibody- and autoantigen-targeted therapies, and therapeutic interference with chronic inflammation and break of tolerance will set the stage for individualized precision medicine, further improvement of outcomes, and eventually cure of AAV (20, 22, 23).

## Pathology

ANCA-associated vasculitidies are systemic necrotizing small-vessel vasculitides, predominantly affecting intraparenchymal small arteries, arterioles, capillaries, venules, and less often medium-sized arteries and veins. In addition, patients with GPA and EGPA display extravascular inflammatory lesions with predilection for the upper and/or lower respiratory tract. Immunohistology discloses few or no immunoglobulin and C3 deposits at inflammatory sites. AAV are hence designated pauci-immune vasculitides (1, 24, 25). Swelling, necrosis, and detachment of endothelial cells are the earliest histomorphological alterations of necrotizing vasculitis. At the vessel wall, both marginating and transmigrating neutrophils undergo apoptosis and karyorrhexis (leukocytoclasia). In the kidney, degranulation of neutrophils induces rupture of glomerular basement membranes and necrosis of adjacent cells, followed by fibrin precipitation. Necrotic debris, fibrin, and proinflammatory factors spill into Bowman’s space. Subsequently, monocytes accumulate and parietal (Bowman’s) epithelial cells proliferate forming crescents. Neutrophils within glomerular lesions also display NETosis, i.e., cellular death characterized by the formation of neutrophil extracellular traps (NETs) (26). Proinflammatory cytokines, chemokines, and complement factors of the alternative pathway locate within inflammatory glomerular lesions. Later stages are characterized by growing influx of monocytes, macrophages, and T- and B-cells. Finally, fibrocellular accumulations progress to fibrotic (sclerotic) lesions. Chronic injury occur more frequently in MPA (27). Notably, renal outcome and risk of relapse correlate with the proportion of sclerotic glomeruli (28, 29) and inversely with the proportion of normal glomeruli (30). In GPA, early extravascular lesions are characterized by the accumulation of neutrophils forming microabscesses. In advanced lesions, geographic patterns of necrosis with peripheral accumulation of macrophages and giant cells are found. Concomitant cellular infiltrates eventually also contain dendritic cells, T- and B-cells, and plasma cells (24, 25). Histopathological classification for extravascular granulomatous lesions in GPA has been proposed (31). Ectopic lymphatic structures have been found in both extravascular granulomatous lesions and glomerulonephritis (32, 33). In EGPA, extravascular and vascular lesions are characterized by eosinophilic infiltration (24, 25).

## Epidemiology and Genetic Background

A yearly incidence between 10 and 20 per million inhabitants and a prevalence of 120 and 140 per million inhabitants has been reported for AAV in Europe and the USA. Prevalence doubling during the last decade has been attributed to improved outcomes (34, 35). Recently, higher incidence of GPA than previously reported has been identified in a UK population (36). A cyclical pattern of occurrence is observed in GPA, but not MPA (37). By contrast, MPA has a higher prevalence than GPA in Japan and China (35). Familial cases are rare in AAV (38). While individual disease-associated alleles carry modest degrees of risk, multiple genetic factors of relatively small effect combine to convey susceptibility to chronic inflammation and autoimmunity (39). Notably, the HLA polymorphism shapes self-epitope specific regulatory T-cell (Treg) responses, thereby mediating protection or causation of autoimmunity (40). Among AAV, GPA displays a remarkable *HLA-DP* association (*p* = 6.2 × 10^−89^) (41). The HLA-DPB1*0401 allele is closely linked to PR3-ANCA^+^ GPA (*p* = 1.2 × 10^−22^) (42, 43). Reduced HLA-DP protein expression is observed in GPA patients with the associated *HLA-DP* allele (44). By contrast, MPO-ANCA^+^ MPA is *HLA-DQ*-associated (*p* = 2.1 × 10^−8^) (41). Furthermore, GPA is associated with polymorphisms of α1-anti-trypsin and PR3 encoding genes (41). In EGPA, association with HLA-DRB1*04 was reported, whereas HLA-DR*13 is underrepresented (45). The *IL10.2* haplotype is associated with ANCA-negative EGPA and increased interleukin (IL)-10 production (46). By contrast, disease-associated PTPN22 *R620W* allele correlates with reduced IL-10 transcription in GPA and MPA (47, 48). Aberrant microRNA expression with dysregulation of genes involved in inflammation and autoimmunity has been reported for various autoimmune diseases (49). In AAV, miRNA expression patterns correlate with renal involvement and steroid doses (50, 51). Upregulated expression of miR-634 induces a proinflammatory phenotype of monocytes in PR3-ANCA^+^ AVV (52). Furthermore, a GPA-specific miRNA expression pattern has been found in nasal tissue differentiating GPA from healthy and disease controls (53). These findings underscore a multifactorial process in which genetic and epigenetic factors play important roles in the genesis of AAV (54–57).

## Barrier Dysfunction in GPA and EGPA

Granulomatosis with polyangiitis is known for its propensity toward necrotizing neutrophilic granulomatous inflammation of the respiratory tract (31). Accordingly, respiratory tract manifestations and flu-like symptoms represent the most frequent initial features and affect virtually all patients with GPA during follow-up in large cohorts (5, 58, 59). Moreover, the so-called “grumbling disease” related to persistent ENT disease activity is observed in many patients being otherwise in clinical remission and despite immunosuppressive treatment (17, 60). Asthma, ENT manifestations, and eosinophilic pulmonary infiltrates also represent the most frequent manifestations in EGPA (7, 61). The nasal mucosa displays a unique gene expression signature with differentially expressed transcripts of antimicrobial peptides, cytokines, extracellular matrix proteins, and molecules important for epithelial barrier integrity in GPA (62, 63). Ciliary motility is severely reduced (64). Chronic nasal carriage of *Staphylococcus aureus* is associated with endonasal activity and relapse in GPA and facilitated by decreased production of human beta-defensin-3 and anomalous cytokine expression pattern of nasal epithelial cells (65–68). Virulence genes, e.g., genes for pore-forming toxins such as leukocidins, may contribute to disease progression and/or relapse in PR3-ANCA^+^ GPA (69). Sensitization to fungi resulting in allergic bronchopulmonary aspergillosis may play a role in the development of EGPA (70). Moreover, exposure to silica is associated with increased risk for AAV (71). Altogether, these findings suggest a link between respiratory barrier dysfunction, infection, and chronic inflammation in GPA and in EGPA (31).

## Cell Death and Chronic Inflammation

Anti-neutrophil cytoplasmic autoantibody-induced pathogenesis is linked to neutrophil activation leading to vascular injury, generation of NETs, apoptosis, and necrosis (25). However, defects in the cell death machinery itself can trigger inflammation and autoimmunity in AAV in extravascular tissues. Dysregulation in neutrophil apoptosis and clearance of apoptotic cells was demonstrated *in vitro* and *in vivo* for GPA (72–74). Apoptotic cells displaying membrane-located PR3 contribute to non-resolving inflammation by disturbing proper clearance (efferocytosis) and subsequent proinflammatory polarization of macrophages (72). Prolonged survival of neutrophils lead to accumulation within inflamed tissue, demonstrated recently *in vivo* in a human transgenic PR3 mouse model (75). Without proper clearing, apoptotic neutrophils undergo secondary necrosis with subsequent release of proinflammatory cytokines, damage-associated molecular patterns (DAMPs), and potential autoantigens (76). Within necrotizing granulomatous inflammation of GPA, release of DAMPs such as high-mobility-group-protein B1 (HMGB1) and IL-33 perpetuate receptor-dependent local auto-amplificatory loops (77). HMGB1, also described as endogenous adjuvant (78), is expressed together with the autoantigen PR3 on the surface of apoptotic neutrophils, potentially contributing to the induction of a pathogenic autoimmune response (79). NETosis is linked to the pathogenesis of AAV by contributing to endothelial damage, stimulation of lymphocytes, and activation of the alternative complement cascade (80, 81). Furthermore, ANCA-induced NET formation has recently been shown to be controlled by caspase-independent form of regulated necrosis, namely necroptosis, in an MPA mouse model (82). Altogether, dysregulation in the cell death machinery can lead to a necro-inflammatory auto-amplification loop (83), a relevant pathogenic feature in AAV and potential target for future treatment strategies.

## Alternative Complement Pathway Activation

Evidence for involvement of the complement system in AAV comes from murine models of MPO-ANCA-induced glomerulonephritis and vasculitis, demonstrating activation of the alternative complement pathway and specifically C5 being essential for disease induction (84, 85). This has been confirmed by proteomic analysis of kidney biopsies (86). Moreover, in the murine model of MPO-ANCA vasculitis, liver-derived C5a is a key mediator (87). MPO- and PR3-ANCA-activated neutrophils elicit C5a release. Subsequent interaction of C5a with the C5a receptor 1 (C5aR1) may represent a proinflammatory amplification loop in AAV (88, 89). Consequently, elevated plasma and serum concentrations of C5a and C3a have been reported in active AAV, especially MPO-ANCA^+^ AAV (90–92). Deposition of complement components is detected in human renal biopsies in AAV (93). Similarly, C5aR1 is expressed scarcely, whereas expression of C5aR2 is upregulated in MPO-ANCA^+^ glomerulonephritis (92). Interestingly, the exact role of C5aR2 has not been clearly defined yet. In the context of AAV, human neutrophil-expressed C5aR2 interacting with C5a seem to play a proinflammatory role (94). However, in the murine model of MPO-ANCA-induced glomerulonephritis, blocking of C5aR1 with a small molecule antagonist (avacopan) is protective, whereas lack of C5aR2 lead to aggravated disease (95). The oral C5aR1-antagonist avacopan yields promising results as glucocorticoid-sparing agent in two randomized phase II clinical trials (NCT01363388 and NCT02222155) (96). In general, tissue expression and function of the anaphylatoxin receptors (C5aR1/2) are still poorly understood, specifically in humans (97). Therefore, it remains to be examined why results regarding tissue expression of C5aR1/2 in MPO-AAV and the function of C5aR2 differ between murine and human studies. Altogether, C5a can be regarded as one of the central players in the pathogenesis of AAV (98). Contribution of the corresponding C5aRs, however, needs to be further investigated.

## Alterations of Effector and Tregs

**Figure 1:**
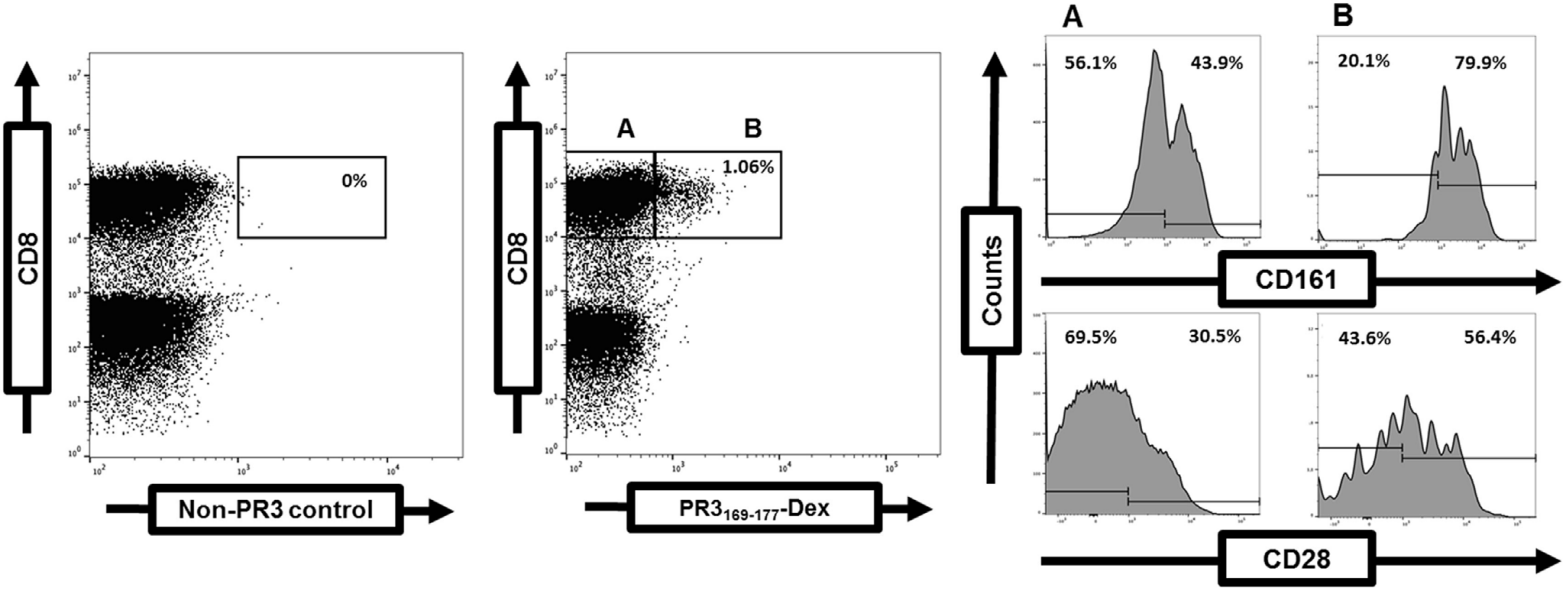
Frequency and phenotype of proteinase 3 (PR3)-specific T-cells determined by flow cytometry. Detection of PR3-specific T-cells within the gated CD3^+^CD8^+^ T-cell population in a GPA-patient (right dot plot). A negative control (left dot plot). Costimulatory CD28 and CD161 expression on gated PR3-specific CD8^+^ T-cells (A) in comparison to PR3-negative CD8^+^ T-cells (B) (4).

Disruption of the balance between regulatory and effector T-cells and accumulation of effector T-cells in tissues with disease propagation favor chronic inflammation and autoimmunity (99). Alterations of the peripheral T-cell compartment have been reported in GPA such as the expansion of circulating effector memory T-cells (T_EM_) including Th1-type CD4^+^ and CD8^+^ T-cell populations lacking costimulatory CD28 expression (CD28^−^), Th17 and Th22 cells, IL-21-producing cells, and CD4^+^CD8^+^ double-positive cells. Conversely, the percentage of circulating naïve T-cells is decreased (4, 100–105). Depletion of circulating Vδ2 T-cells, mucosal-associated invariant T-cells, and innate lymphoid T-cell subsets have been observed (106, 107). Remission is associated with a shift toward circulating total Th2, Th9, and Treg populations in GPA (105). Notably, the cytokine profile of PR3-specific T-cells is skewed toward Th2-type, Th17, and Th22 cells independent of disease activity (102, 108, 109). PR3-specific T-cells are CCR7^−^CD45RA^−^ T_EM_ with either “intermediate” CD28^+^CD27^−^ or “early” CD28^+^CD27^+^ phenotype (4, 108, 109). They may express the costimulatory c-type lectin CD161 (Figure 1).

Percentages of total circulating T_EM_ decrease during active disease, suggestive of T_EM_ migration toward inflamed sites (101). Accordingly, CD28^−^ T_EM_ are abundant in bronchoalveolar fluid, inflammatory lesions, and, during renal activity, in urine (100, 110, 111). In EGPA, the frequency of circulating total Th2 and Th17 cell populations is increased, whereas lower numbers of Treg are detected (112–114). Phenotype and function of Treg are altered and impaired, respectively, in AAV (115, 116). Expansion of circulating and tissue-resident CD28^−^ T_EM_ lacking IL-2 production could favor Treg dysfunction in GPA (100). In line with an antigen-driven pathogenesis, oligoclonal T-cell proliferation has been reported in AAV previously (117, 118). Expansion of circulating CD28^−^ T-cells has been attributed to latent cytomegalovirus (CMV) infection in GPA (119). However, concomitant cellular CMV- and Epstein–Barr virus (EBV)-infection rather than sole CMV or EBV infection is associated with expansion of CD28^−^ T_EM_ (4). Transcriptome analysis suggests pathogen (*S. aureus*, EBV, and others) and inflammation (cytokine and chemokine)-driven alterations of the peripheral T-cell compartment linking autoimmune disease and infection-triggered pathology in GPA (4).

## ANCA Immunoglobulins

Myeloperoxidase- and PR3-ANCA are hallmarks of autoimmune vasculitis in AAV (Table S1 in Supplementary Material) (23, 120, 121). Functionally, numerous *in vitro* studies demonstrate IgG MPO- and PR3-ANCA interaction with neutrophils and monocytes. Interaction between circulating ANCA and neutrophils and monocytes causing their activation, PR3- and MPO-translocation to the cellular surface, microvascular adherence, vascular inflammation, and cell death is known as ANCA-cytokine-sequence theory (122). Notably, PR3- and MPO-ANCA display differences in neutrophil activation, e.g., respiratory burst (123, 124). In known or suspected AAV, the revised consensus on the use of ANCA testing now recommends high-quality immunoassays as primary screening method (13). Regarding IgG MPO-ANCA, epitope specificity determines pathogenicity, mainly by identifying an epitope of MPO (anti-MPO_447-459_) that is exclusively linked to active MPA (125). IgG MPO-ANCA can be masked by a fragment of ceruloplasmin (125). Thus, true ANCA-negative vasculitis is considered as being less than 10% using contemporary ANCA tests (13, 121). In EGPA, IgA MPO-ANCA can occur together with IgG MPO-ANCA, but seem of less value as a biomarker (126). Of note, IgG, IgA, and IgM MPO-ANCA representing low-titer natural autoantibodies are found in healthy donors (126–128). With respect to PR3-ANCA, especially IgG isotypes (122), but IgA and IgM as well, have been linked to GPA. IgA PR3-ANCA are observed in about a quarter of GPA patients, being less prevalent in severe renal disease (129). Transient and recurring presence of IgM PR3-ANCA is reported in proportions of two large cohorts of patients with GPA or MPA and associated with a higher rate of alveolar hemorrhage (130). Similar to MPO-ANCA, IgG, IgA, and IgM PR3-ANCA are detected in healthy donors. However, the epitope specificity of natural ANCA in healthy donors differs from that of pathogenic ANCA in AAV (125, 127–129). Several studies indicate that ANCA and other autoantibodies present in AAV recognize targets apart from MPO and PR3 (Table S1 in Supplementary Material). Some of these studies though still lack confirmatory data or functional relevance.

## ANCA Glycosylation

Altered Fc N-glycosylation patterns of IgG, differing between proinflammatory (agalactosylated) and antiinflammatory (galactosylated and sialylated) IgG antibodies (131), may contribute to the pathogenicity of IgG-ANCA and be a useful biomarker. Agalactosylation of total IgG has been associated with MPA, GPA, and EGPA (132). Instead, sialylation of IgG PR3-ANCA inversely correlates with disease activity and ROS production by neutrophils in GPA (133). Employing mass spectroscopy, reduced galactosylation and sialylation of IgG1 PR3-ANCA or total IgG was confirmed (134, 135). IgG-ANCA bind *via* their Fc part to FcγRIIa and FcγRIIIb, thereby co-operating with the antigen-binding site in neutrophil activation (136). Alteration of IgG-ANCA glycosylation attenuates experimental ANCA-induced glomerulonephritis (137).

## Animal Models of ANCA-Induced Vasculitis and Extravascular Granulomatosis

While murine models demonstrated convincing evidence for MPO-ANCA-induced glomerulonephritis and vasculitis (138), it has proven extremely difficult to show pathogenicity of PR3-ANCA *in vivo* (139). Transfer of human hematopoietic stem cells and anti-PR3 antibodies in NOD-SCID-IL-2Rγ^−/−^ mice induced disease manifestations partially resembling systemic vasculitis (140), giving a hint of PR3-ANCA pathogenicity. A transgenic mouse model expressing human PR3 displays enhanced neutrophil survival and accumulation reflecting aspects of granulomatous inflammation in GPA (75). Besides active immunization and passive transfer of autoimmunity (141), transplantation of GPA tissue into immunodeficient mice provides evidence that fibroblasts induce nasal cartilage and bone destruction seen in GPA (142).

## Autoreactive Circulating and Lesional B Lymphocytes

Increased proportion of circulating matured B-cells recognizing PR3 in PR3-ANCA^+^ AAV (143), presence of ectopic lymphoid structures and autoreactive B-cells in inflamed tissue of GPA (144, 145) and glomerulonephritis (32) provide a rationale for targeting B-cells. B-cell depletion proves to be an efficient therapy in AAV (146–148). Although IL-10-producing regulatory B-cells (Bregs) and CD24^high^ CD38^high^ Bregs, respectively, are reduced in AAV (149–151), their role in AAV remains to be further defined as such circulating cells are probably removed by B-cell-depleting therapies. Altogether, as possible scenario for ANCA-mediated pathogenesis, one could envision that B-cells producing natural autoantibodies which recognize PR3, MPO, or other antigens are selected in an inflamed microenvironment and mature with the help of autoantigen-specific T-cells. Subsequently, this could lead to the emergence and survival of pathogenic plasma cells (152) producing proinflammatory ANCA.

## Conclusion

Much progress has been achieved in elucidating pathogenetic mechanisms in AAV, especially regarding the discovery of links between genetic predispositions and ANCA as well as deciphering mostly deleterious functions of PR3- and MPO-ANCA by *in vitro* and in case of the latter by *in vivo* studies. Future research projects should, for instance, focus on investigating the role of microbes (bacteria and viruses) in the etiology of AAV. Altogether, a better understanding of the complex interplay between endogenous and exogenous factors contributing to pathogenic PR3- and MPO-ANCA may support the development of individualized therapies.

## Author Contributions

PL, AM, and AK substantially contributed to this review with regard to content and structure of the manuscript. All other authors listed have made direct and/or intellectual contribution to sections according to their area of expertise. All authors approved the manuscript for publication.

## Conflict of Interest Statement

The authors declare that the research was conducted in the absence of any commercial or financial relationships that could be construed as a potential conflict of interest.
