# Supplementary material for: Pathogenetic and Clinical Aspects of Anti-Neutrophil Cytoplasmic Autoantibody-Associated Vasculitides
Source: Front Immunol. 2018 Apr 9;9:680. doi: 10.3389/fimmu.2018.00680 (PMC5900791; doi:10.3389/fimmu.2018.00680)
Supplement: Supplementary file 1 [file table_1.docx]

Supplementary Material

Pathogenetic and clinical aspects of ANCA-associated vasculitides

Peter Lamprecht*, Anja Kerstein, Sebastian Klapa, Susanne Schinke, Christian M. Karsten, Xinhua Yu, Marc Ehlers, Jörg T. Epplen, Konstanze Holl-Ulrich,Thorsten Wiech, Kathrin Kalies, Tanja Lange, Martin Laudien, Tamas Laskay, Timo Gemoll, Udo Schumacher, Sebastian Ullrich, Hauke Busch, Saleh Ibrahim, Nicole Fischer, Katrin Hasselbacher, Ralph Pries, Frank Petersen, Gesche Weppner, Rudolf Manz, Jens Y. Humrich, Relana Nieberding, Gabriela Riemekasten, Antje Müller

**Correspondence:** Corresponding Author: peter.lamprecht@uksh.de

# Supplementary Data

none

# Supplementary Figures and Tables

| **ANCA specificities** | | **linked to:** | **references** |
| --- | --- | --- | --- |
| **MPO** | | | |
| IgG MPO-ANCA | 25 linear and conformational MPO epitopes | MPA (12 anti-MPO antibodies), EGPA, healthy donors | (1–3) |
| IgA MPO-ANCA | epitope(s) unknown | EGPA, healthy donor | (4) |
| IgM MPO-ANCA | epitope(s) unknown | healthy donors | (5) |
| **PR3, LAMP2, Pentraxin-3, Lactoferrin** | | | |
| IgG PR3-ANCA (IgG1, IgG3, IgG4) | linear and conformational PR3 epitopes | GPA, healthy donors | (1, 3, 6, 7) |
| IgA PR3-ANCA | epitope(s) unknown | GPA, healthy donor | (8) |
| IgM PR3-ANCA | epitope(s) unknown | GPA, MPA, healthy donor | (5) |
| IgG complementary PR3-ANCA | 97mer of the anti-sense DNA sequence of PR3 (homology to microbial peptides) | PR3-ANCA disease | (9–11) |
| hLAMP2-ANCA | 9mer peptide, (100% homology to bacterial FimH) | AAV, including ANCA-negative AAV | (12–14) |
| Pentraxin-3-ANCA | epitope(s) unknown | MPA, GPA, EGPA, ANCA-negative AAV | (15) |
| IgG Lactoferrin-ANCA | epitope(s) unknown | EGPA | (16) |
| **other autoantigens** | | | |
| anti-plasminogen IgG, anti-tissue plasminogen activator IgG | not fully defined, conformational epitopes are likely | AAV | (10, 17) |
| anti-TMEM9b, anti-tetraspanin 7 | epitopes unknown | GPA | (18) |
| anti-Endothel IgG | lamin A, vimentin, α-enolase, FUBP2, PDIA3 (epitopes unknown) | MPA, healthy donor | (19) |
| anti-progranulin IgG | epitope(s) unknown | autoimmune diseases, including AAV | (20) |

**Table 1.** Summary of studies demonstrating associations between specificities of ANCA or other autoantibodies and autoimmune vasculitis.

**References**

1. Prüßmann J, Prüßmann W, Recke A, Rentzsch K, Juhl D, Henschler R, et al. Co-occurrence of autoantibodies in healthy blood donors. Exp Dermatol 2014;23:519–21. doi:10.1111/exd.12445.
2. Roth AJ, Ooi JD, Hess JJ, van Timmeren MM, Berg EA, Poulton CE, et al. Epitope specificity determines pathogenicity and detectability in ANCA-associated vasculitis. J Clin Invest 2013;123:1773–83. doi:10.1172/JCI65292.
3. Cui Z, Zhao M, Segelmark M, Hellmark T. Natural autoantibodies to myeloperoxidase, proteinase 3, and the glomerular basement membrane are present in normal individuals. Kidney Int 2010;78:590–7. doi:10.1038/ki.2010.198.
4. Oommen E, Hummel A, Allmannsberger L, Cuthbertson D, Carette S, Pagnoux C, et al. IgA antibodies to myeloperoxidase in patients with eosinophilic granulomatosis with polyangiitis (Churg-Strauss). Clin Exp Rheumatol 2017;35:98–101. doi:10.1007/128.
5. Finnern R, Bye JM, Dolman KM, Zhao MH, Short A, Marks JD, et al. Molecular characteristics of anti-self antibody fragments against neutrophil cytoplasmic antigens from human V gene phage display libraries. Clin Exp Immunol 1995;102:566–74.
6. Brouwer E, Tervaert JW, Horst G, Huitema MG, van der Giessen M, Limburg PC, et al. Predominance of IgG1 and IgG4 subclasses of anti-neutrophil cytoplasmic autoantibodies (ANCA) in patients with Wegener’s granulomatosis and clinically related disorders. Clin Exp Immunol 1991;83:379–86.
7. Mulder a H, Stegeman C a, Kallenberg CG. Activation of granulocytes by anti-neutrophil cytoplasmic antibodies (ANCA) in Wegener’s granulomatosis: a predominant role for the IgG3 subclass of ANCA. Clin Exp Immunol 1995;101:227–32. doi:10.1111/j.1365-2249.1995.tb08343.x.
8. Kelley JM, Monach P a, Ji C, Zhou Y, Wu J, Tanaka S, et al. IgA and IgG antineutrophil cytoplasmic antibody engagement of Fc receptor genetic variants influences granulomatosis with polyangiitis. Proc Natl Acad Sci 2011;108:20736–41. doi:10.1073/pnas.1109227109.
9. Pendergraft WF, Preston G a, Shah RR, Tropsha A, Carter CW, Jennette JC, et al. Autoimmunity is triggered by cPR-3(105–201), a protein complementary to human autoantigen proteinase-3. Nat Med 2004;10:72–9. doi:10.1038/nm968.
10. Bautz DJ, Preston GA, Lionaki S, Hewins P, Wolberg AS, Yang JJ, et al. Antibodies with dual reactivity to plasminogen and complementary PR3 in PR3-ANCA vasculitis. J Am Soc Nephrol 2008;19:2421–9. doi:10.1681/ASN.2008030270.
11. Tadema H, Kallenberg CGM, Stegeman C a., Heeringa P. Reactivity against complementary proteinase-3 is not increased in patients with PR3-ANCA-associated vasculitis. PLoS One 2011;6:1–5. doi:10.1371/journal.pone.0017972.
12. Kain R, Tadema H, McKinney EF, Benharkou a., Brandes R, Peschel a., et al. High Prevalence of Autoantibodies to hLAMP-2 in Anti-Neutrophil Cytoplasmic Antibody-Associated Vasculitis. J Am Soc Nephrol 2012;23:556–66. doi:10.1681/ASN.2011090920.
13. Roth AJ, Brown MC, Smith RN, Badhwar AK, Parente O, Chung H chul, et al. Anti-LAMP-2 antibodies are not prevalent in patients with antineutrophil cytoplasmic autoantibody glomerulonephritis. J Am Soc Nephrol 2012;23:545–55. doi:10.1681/ASN.2011030273.
14. Peschel A, Basu N, Benharkou A, Brandes R, Brown M, Dieckmann R, et al. Autoantibodies to hLAMP-2 in ANCA-negative pauci-immune focal necrotizing GN. J Am Soc Nephrol 2014;25:455–63. doi:10.1681/ASN.2013030320.
15. Simon A, Subra JF, Guilpain P, Jeannin P, Pignon P, Blanchard S, et al. Detection of anti-pentraxin-3 autoantibodies in ANCA-associated vasculitis. PLoS One 2016;11:1–13. doi:10.1371/journal.pone.0147091.
16. Shida H, Nakazawa D, Tateyama Y, Miyoshi A, Kusunoki Y, Hattanda F, et al. The presence of anti-lactoferrin antibodies in a subgroup of eosinophilic granulomatosis with polyangiitis patients and their possible contribution to enhancement of neutrophil extracellular trap formation. Front Immunol 2016;7:1–7. doi:10.3389/fimmu.2016.00636.
17. Berden AE, Nolan SL, Morris HL, Bertina RM, Erasmus DD, Hagen EC, et al. Anti-Plasminogen Antibodies Compromise Fibrinolysis and Associate with Renal Histology in ANCA-Associated Vasculitis. J Am Soc Nephrol 2010;21:2169–79. doi:10.1681/ASN.2010030274.
18. Thurner L, Müller A, Cérutti M, Martin T, Pasquali J-L, Gross WL, et al. Wegener’s granuloma harbors B lymphocytes with specificities against a proinflammatory transmembrane protein and a tetraspanin. J Autoimmun 2011;36:87–90. doi:10.1016/j.jaut.2010.09.002.
19. Régent A, Lofek S, Dib H, Bussone G, Tamas N, Federici C, et al. Identification of target antigens of anti-endothelial cell antibodies in patients with anti-neutrophil cytoplasmic antibody-associated vasculitides: A proteomic approach. Clin Immunol 2014;153:123–35. doi:10.1016/j.clim.2014.03.020.
20. Thurner L, Preuss K-D, Fadle N, Regitz E, Klemm P, Zaks M, et al. Progranulin antibodies in autoimmune diseases. J Autoimmun 2013;42:29–38. doi:10.1016/j.jaut.2012.10.003.

## Supplementary Figures

none
